# Unidirectional Enhanced Dipolar Emission with an Individual Dielectric Nanoantenna

**DOI:** 10.3390/nano9040629

**Published:** 2019-04-18

**Authors:** Tianyue Zhang, Jian Xu, Zi-Lan Deng, Dejiao Hu, Fei Qin, Xiangping Li

**Affiliations:** Guangdong Provincial Key Laboratory of Optical Fiber Sensing and Communications, Institute of Photonics Technology, Jinan University, Guangzhou 510632, China; yusheng370@163.com (J.X.); zilandeng@jnu.edu.cn (Z.-L.D.); dejiaohu@jnu.edu.cn (D.H.); xiangpingli@jnu.edu.cn (X.L.)

**Keywords:** unidirectional emission, fluorescence, hybrid nanoantenna

## Abstract

Light manipulation at the nanoscale is the vanguard of plasmonics. Controlling light radiation into a desired direction in parallel with high optical signal enhancement is still a challenge for designing ultracompact nanoantennas far below subwavelength dimensions. Here, we theoretically demonstrate the unidirectional emissions from a local nanoemitter coupled to a hybrid nanoantenna consisting of a plasmonic dipole antenna and an individual silicon nanorod. The emitter near-field was coupled to the dipolar antenna plasmon resonance to achieve a strong radiative decay rate modification, and the emitting plasmon pumped the multipoles within the silicon nanorod for efficient emission redirection. The hybrid antenna sustained a high forward directivity (i.e., a front-to-back ratio of 30 dB) with broadband operating wavelengths in the visible range (i.e., a spectral bandwidth of 240 nm). This facilitated a large library of plasmonic nanostructures to be incorporated, from single element dipole antennas to gap antennas. The proposed hybrid optical nanorouter with ultracompact structural dimensions of 0.08 λ^2^ was capable of spectrally sorting the emission from the local point source into distinct far-field directions, as well as possessing large emission gains introduced by the nanogap. The distinct features of antenna designs hold potential in the areas of novel nanoscale light sources, biosensing, and optical routing.

## 1. Introduction

Controlling light emissions from nanoemitters has key merit in many applications, such as biological sensing and imaging, surface-enhanced spectroscopy, light-emitting devices, and on-chip integrated sources [1,2]. Plasmonic nanostructures, with the ability to manipulate the excitation and emission processes of the localized emitters, function as transducers between far- and near-field light signals working at the optical frequency known as optical nanoantennas [3]. Owing to the engineered localized density of photonic states introduced by the plasmon resonance, plasmonic nanostructures can concentrate the optical electric field, alter the de-excitation pathways, change the emission polarization, and redirect the emission intensity, leading to various functionalities like fluorescence enhancement, lifetime shortening, spectrum reshaping, and direction steering [4,5,6,7]. Because of the nature of resonant enhancement, the far-field angular emission of the emitter-antenna coupling system is determined strongly by the antenna modes [8,9], whilst the most commonly used nanoantennas (e.g., metallic nanoparticles and gap antennas) possess dominant dipolar plasmon resonances with omnidirectional characteristics, which are disadvantageous for directional emission control.

Many antenna designs have been demonstrated to effectively redirect emissions to target directions by using complex, multicomponent plasmonic nanostructures, such as optical Yagi-Uda nanoantennas [10,11], aperture-groove structures [6,12,13], and nanoantenna arrays [14]. Metallic patch [5,15] and planar antennas [16,17] have shown out-of-plane unidirectionality. Single-element antennas such as V- and U-shaped antennas also provide directional light control capabilities arising from multipolar interference [9,18,19,20]. However, the footprints of these antennas need to be considerably large enough for optical phase accumulation, or to support multipolar plasmon modes. Ultracompact nanoantennas consisting of a pair of interacting plasmonic nanoparticles have been reported to exhibit a directionality effect. However, this is only true for certain well-defined emitter-antenna configurations, and the unidirectional emission only works well in a narrow wavelength bandwidth [21]. Shaping the light radiation to be unidirectional in parallel with the high emission enhancement is still a challenge for ultracompact nanoantennas far below the subwavelength dimensions.

The recent emerging field of all-dielectric nanoantennas offers exciting opportunities for light manipulation, with those structures supporting both electric and magnetic Mie resonances [22]. Studies have shown that the all-dielectric nanoantennas exhibit a unique capability to create unidirectional radiation due to the far-field interference of the induced multipolar antenna modes excited by the localized nearby emitter [23,24,25]. However, their low field enhancement and the limited radiation enhancement hamper their wide use in spectroscopy and imaging. Hybrid metal-dielectric nanoantennas combine the advantages of both manifest rapid development in various applications, including linear and nonlinear control of light scattering, and the control of near-field characteristics [26,27,28,29,30,31,32]. Recent demonstrations using the hybrid metal-dielectric antennas for unidirectional emission are mostly implemented in the near-IR range [26,29]. A highly compact antenna design allows for effective emission amplification and, with the optical signals selectively radiated in a certain spatial direction, has been seldom reported in the visible regime.

In this article, we theoretically demonstrate that the dipolar emission pattern can be reshaped to be highly unidirectional by placing a conventional plasmonic antenna–nanoemitter system in the vicinity of an individual silicon nanorod. In this hybrid system, the emitter near-field coupled to the dipolar antenna plasmon resonance (i.e., radiative decay engineering), then the emitting plasmon pumped the multipoles in the silicon nanorod (i.e., emission redirection). The silicon nanorod thus behaved as a unidirectional scattering element that favored forward radiation front-to-back ratios of up to approximately 30 dB. A variety of plasmonic dipole antennas were exploited and integrated to realize directive emissions in the hybrid fashion. Moreover, we created an optical router consisting of plasmonic dimer antennas and the silicon nanorod, with a device footprint as small as 0.08 λ^2^. In addition to the large emissions gain introduced by the nanogap, the multi-resonant hybrid antennas enabled the emission of the local light source to be remarkably directed and switched between two distinct directions based on its color. With such unique features, it shows great potential for applications such as quantum single photon sources and integrated optical circuits.

## 2. Materials and Methods

The three-dimensional finite-difference time-domain method (lumerical FDTD 2019a), was employed to calculate the local electromagnetic fields of the nanoantennas, fluorescence enhancement of the emitter-antenna coupled system, and the corresponding far-field radiation patterns [33]. A classical point dipole source representing a single emitter was implemented in the FDTD calculations. The presence of the nanoantennas modifies the spontaneous emission rate of the emitter through the Purcell effect. Purcell factor (*PF*), which characterizes the enhancement of the radiative decay rate, is numerically calculated by the relationship $PF=P_{rad}/P_{rad}^{0}$, where $P_{rad}$ and $P_{rad}^{0}$ are the radiated power out of the dipole-antenna system and the radiated power of the dipole in a homogeneous environment, respectively [34]. Antenna efficiency, which is the power that reached the far field ($P_{rad}$) divided by the total emitting power of the dipole source ($P_{tot}$), represents the quantum efficiency of the optical antenna. This is calculated using $\eta_{a}=P_{rad}/P_{tot}$, where $P_{tot}$ was obtained by integrating the Poynting vector over closed surfaces containing the dipolar source only [35,36].

The far-field angular patterns of the emitter-antenna system in the free space were obtained by recording the fields component in a closed box monitor, and near-to-far-field transformation (NTFF) was performed to these fields. When the substrate was included, we first recorded the near-field components at the monitor with sufficiently large surfaces (15 × 15 µm^2^) 50 nm below the interface and projected the near-field data to the far field [33,37,38].

To gain the insight of the unidirectional property of the hybrid antenna, Cartesian multipole expansion based on the induced multipole moments was performed. This served as the indication of which modes were effectively excited and contribute to the unidirectional emission. The Cartesian electric dipole (ED) and magnetic dipole (MD) moments can be calculated as [24,39]

$$\vec{P}=\frac{i}{\omega }\int \vec{J}(\vec{r})d^{3}\vec{r}$$

$$\vec{M}=\frac{1}{2c}\int \vec{r}\times \vec{J}(\vec{r})d^{3}\vec{r}$$

where $\vec{J}(\vec{r})=-i\omega \varepsilon_{0}(\varepsilon_{r}-1)\vec{E}$ is the induced current in the structure and $\vec{r}$ is the position vector with the origin at the center of the hybrid antenna.

In all the calculations, a perfectly matched layer boundary was used, and the mesh size in the vicinity of the antenna was set to 1 nm for all cases. The dielectric function of gold was taken from the Johnson and Christy data [40], and the refractive indices of silicon was taken from Palik [41].

## 3. Results

**Figure 1:**
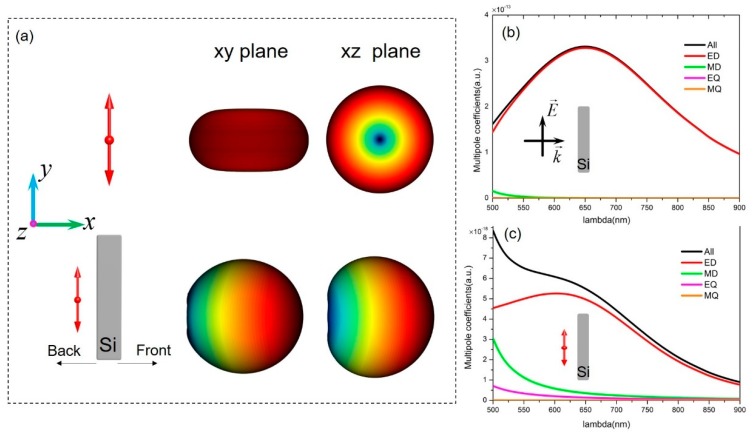
Principles of a single silicon nanorod used as a directive scattering element. (**a**) Sketch of far-field radiation reshaping an electric dipole emitter in the vicinity of a silicon nanorod in free space. The size of the nanorod is 350 (length) × 50 (width) × 120 nm (height), and the point emitter is 30 nm away from the silicon surface. (**b**) The Cartesian multipole decomposition of the silicon nanorod based on the internal induced currents under plane wave excitation and (**c**) the multipole decomposition under the dipole excitation. Only the non-negligible multipole coefficients are shown.

We first investigated the single electric point dipole in the vicinity of the individual silicon nanorod. The broadband dipole source mimicked the conventional ED-type nanoemitters, such as quantum dots and fluorescent dyes. In Figure 1a, the red double arrow in the schematic indicates the orientation of the dipole axis (along the y-axis), and the corresponding three-dimensional (3D) far-field radiation pattern for a single dipole in free space is shown to be omnidirectional. When the electric dipole is placed close to the silicon nanorod, the high refractive index silicon nanorod permits its intrinsic Mie resonances to be locally excited. Although conventional silicon nanoparticles have been shown to support Mie resonances when illuminated with a plane wave, the dipole excitation is physically different. Dipole excitation provides more degrees of freedom to control the excited modes. This has been proven by numerous studies of emission characteristics of the coupled systems, in which various dipole–antenna configurations were extensively investigated [1,4,8,9,35,36]. In the following content, we will show that the interplays between the excited Mie eigenmodes inside the silicon nanorod and the local emitter boost emission directivity in an unprecedented way.

To gain insight on the origin of emission directivity, we first performed the Cartesian multipole expansion of the total scattering response based on the induced current of the silicon nanorod. The designed silicon nanorod with a length of 350 nm (L), a width of 50 nm (W), and a height of 120 nm (H) shown in Figure 1b supports the dominant dipolar electric mode in the visible wavelength region. For comparison, multipole expansion was also performed under dipole excitation in order to provide a more accurate picture of the system. A similar result is presented in Figure 1c (except for the weak excitation of the MD mode), and we can come to the same conclusion that the ED mode of the silicon nanorod offers a pronounced contribution to the unidirectional property. Two coherent dipoles, separated by an appropriate distance and with their phase and amplitude well-matched, were previously reported to produce unidirectional emissions [19,37]. In the present case, due to the far-field interference between the coherent electromagnetic fields produced by the source dipole and the induced ED in the silicon nanorod, most of the emission power was directed forward, leading to an ultrahigh front-to-back-ratio (F/B ratio). Reported studies have used a single silicon disk to achieve the functionality of unidirectional emissions. However, the operating wavelength lies at 1.1 µm with a disk diameter of over 600 nm in order to utilize the interference between the excited magnetic dipolar mode and electric quadrupolar mode [26,29]. Herein, we utilized a much smaller sized silicon nanorod to realize the directivity in the visible spectrum, thus dramatically reducing the nanoantenna dimensions.

**Figure 2:**
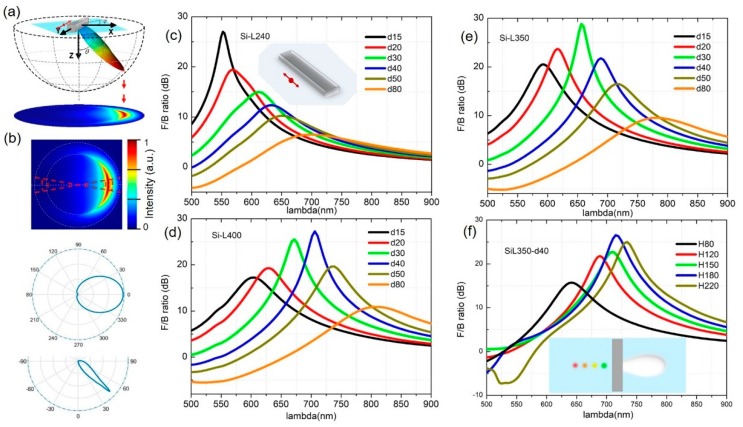
Far field angular distributions and F/B ratios of the nanoantenna on a substrate. (**a**) 3D plot of the far-field radiation into the lower half space and its corresponding 2D projections in BFP. (**b**) Top: BFP image with red dashed lines indicating the intensity integrating area in relation to the calculated F/B ratio. Bottom: Azimuthal polar plot (φ = 0 to 360°) at the angle of maximum emission (θc = 43.6°) and the cross section of the emission as a function of the polar angle (θ). F/B ratios as a function of wavelength for a silicon rod length of (**c**) 240, (**d**) 400, and (**e**) 350 nm for different emitter–silicon separations. The inset in (**c**) shows the configuration of the emitter-antenna system in the calculations. The light grey plane indicates the substrate interface. (**f**) The F/B ratio as a function of wavelength for different silicon rod heights. The red double arrow indicates the dipole emitter. The inset in (**f**) shows a simple picture of the on-chip integration of nanosources of different emission colors to provide unidirectional white light in the output of the device.

Figure 2a highlights the features of a 3D far-field intensity distribution when a substrate is included, with an azimuthal angle (φ) and polar angle (θ). This allows us to evaluate the emission in every direction. In practical experiments, back focal plane (BFP) imaging is commonly used to measure the far-field angular distribution in the Fourier plane. Therefore, the 3D plot was transformed into 2D projections. The F/B ratio is defined as the ratio of light intensity radiated in the positive x-direction to that in the negative direction according to the calculated BFP patterns. In order to quantify the directional performance of the hybrid system [19,42], the following formula was used.

$$F/B=10\log_{10}\frac{\int_{\theta_{c}-\delta }^{\theta_{c}+\delta }\int_{-\delta }^{\delta }S(\varphi ,\theta )\sin\theta d\theta d\varphi }{\int_{\theta_{c}-\delta }^{\theta_{c}+\delta }\int_{\pi -\delta }^{\pi +\delta }S(\varphi ,\theta )\sin\theta d\theta d\varphi }$$

where S(φ,θ) is the intensity of the BFP image and $\delta =10^{\circ }$ is the half angle for the integration cone. It has been recognized that the light emits predominantly into the high-index medium when there is an interface. The emission intensity peaks at around $\theta =\theta_{c}=\arcsin(1/n_{glass})$ for the air-glass interface in our case. Therefore, the solid angle over which the integrals are calculated is limited by the critical angle (θ_c_). The considered integral regions are enclosed in red dash lines in Figure 2b. The azimuthal polar plot (φ = 0 to 360°) at the angle of maximum emission (θ_c_ = 43.6°) and the cross section of the emission pattern as a function of the polar angle (θ) are also represented.

The emitter near-field couples to the electric dipolar resonance of the silicon nanorod. This, in turn, couples to the radiation field with a certain far-field angular pattern. The dimension of the silicon nanorod determines the spectral features of the induced ED mode, while the emitter–antenna distance has a strong influence on the phase relationship between the source dipole and the induced electric dipole in silicon. Hence, under the proper circumstances of balancing the relative amplitudes and the phases, the backward emission can be eliminated. F/B ratio values derived from the BFP images for different rod length situations are shown in Figure 2c–e as a function of wavelength, varying the distances between the dipole and the silicon rod surface. It was also found that the height and the width of the silicon nanorod largely affected the magnetic component of the resonant multipoles. Thus, in order to inhibit the magnetic contribution and make the antenna design compact, we fixed the width of the silicon nanorod to be 50 nm and the height to be 120 nm in the rest of the calculations.

Importantly, the silicon nanorod supports a remarkable broad wavelength range of forward directivity. For instance, when the silicon nanorod is 350 nm in length, a large F/B ratio (>10 dB) can be achieved from 540 to 780 nm (i.e., a bandwidth of approximately 240 nm) with the appropriate emitter–silicon separation tunable from 15–80 nm. In practical applications, the local point source can be a quantum emitter, a fluorescent dye, or other nanosources; these usually have a narrow emission band. Our results show that, for different-colored sources, there always exists an optimal position at which the directivity is at maximum. Based on such observations, a simple picture of integrating multi-sources to the silicon nanorod is shown in the inset of Figure 2f. By placing each source at the best position, accordingly, the output of the whole system could be made to be unidirectional white light.

**Figure 3:**
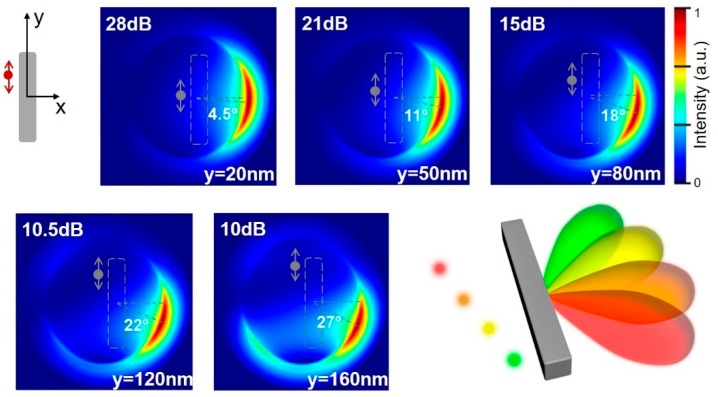
The influence of lateral displacement on radiation direction. Angular emission patterns with different dipole y-positions at 660 nm. The F/B values and the deflection angle of the emission lobe are also shown. The cartoon illustrates a brief description of the possible implementation of an integrated nanodevice for angle-resolved color routing.

We then investigated the effect of the relative spatial configuration of the dipole source and the silicon nanorod on emission directivity. Particularly, we showed that the far-field angular patterns were sensitive to lateral displacement of the emitter position in y-direction, as displayed in Figure 3. In all these cases, the dipole emitter was placed 30 nm away from the silicon (i.e., a length of 350 nm), and the optimal F/B ratio always occurred at 660 nm. As the lateral distance was increased along the y-direction, the forward emission features remained unchanged, but the maximal azimuthal angle (φ) of the angular patterns increased accordingly. When the displacement was 20 nm, the forward emission main lobe was observed to be shifted to 4.5°. With further enlargement of the offset to 80 nm, the main lobe was redirected to φ_max_ = 18°. The strong directivity (F/B > 10 dB) was even preserved at a y-offset of 160 nm. This means that the unidirectional effect can be realized for a large working distance. Such beam redirection characteristics are promising for applications such as optical interconnects. One possible implementation shown in Figure 3 is to integrate different colored nanosources onto a single silicon nanorod with various relative configurations (i.e., proper x-position and y-displacement), and the resulting device will provide multi-color emissions in different angular directions. This is especially applicable for on-chip optical communications.

**Figure 4:**
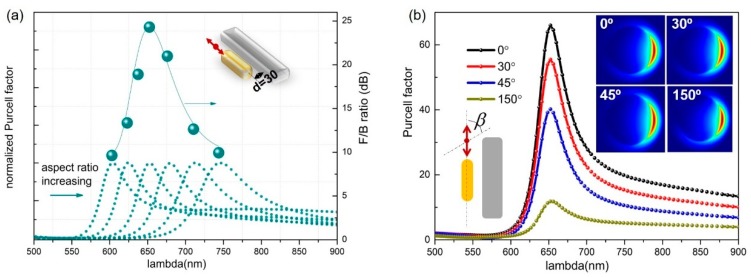
(**a**) The PF and F/B ratios for hybrid antennas consisting of gold and silicon nanorods. The red double arrow indicates the dipole emitter. For all the calculations, the emitter was placed 10 nm away from the gold. The inset shows the configuration of the emitter-hybrid-antenna system in the FDTD calculations. (**b**) The effect of different dipole orientations on PFs and far-field radiation patterns. The insets show the far-field patterns are kept to be unidirectional for different dipole orientation conditions.

Our initial investigations, starting from the single dipole emitter nearby the silicon nanorod, helps in the creation of guidelines for more antenna designs to achieve unidirectional emissions. In fact, the dipole emitter can be replaced by any other light sources with dipolar emission behavior. For instance, when a nanoemitter is coupled to a small gold nanorod (AuNR) in an end-to-end fashion, the emission presents a dipolar nature. As shown in Figure 4, the gold nanorod with a diameter of 40 nm was positioned in parallel with the silicon rod (with a length of 350 nm) and 30 nm away from the silicon surface. Changing the length of the gold nanorod from 80 to 130 nm, which varies the aspect ratio of gold nanorods, leads to the resonance wavelength of the emitter–AuNR coupled system spanning 600–750 nm. Although the point emitter is a broadband local source, the strong resonant coupling with the AuNR makes the total emission dominant at the plasmonic mode. We calculated the F/B ratio at the resonance wavelength for each AuNR length case of the hybrid antenna system and plotted the F/B values in discrete points, as shown in Figure 4a. Visualizing these data points by a connecting line gives us a perfect reproduction of the trend corresponding to a single emitter placed 30 nm away from the 350 nm long silicon nanorod (see the green line in Figure 2e).

We further investigated the effect of the dipole orientations. We define β to be the angle between the dipole axis and the long axis of AuNR. It is evident that β = 0 represents the most efficient coupling of the dipole source and AuNR, such that the configuration provides highest PF. Other configurations did not change the spectral position of maximal PF since, in all the cases, the longitudinal dipolar mode of AuNR was excited. The far-field emission patterns are presented in the inset of Figure 4b to show that the dipolar omnidirectional emission characteristics were reshaped to be unidirectional for different dipole orientation situations. Such observations give us clear evidence that, irrespective of the dipole orientations, the dipolar mode of the AuNR makes the deterministic contribution to the unidirectional property.

**Figure 5:**
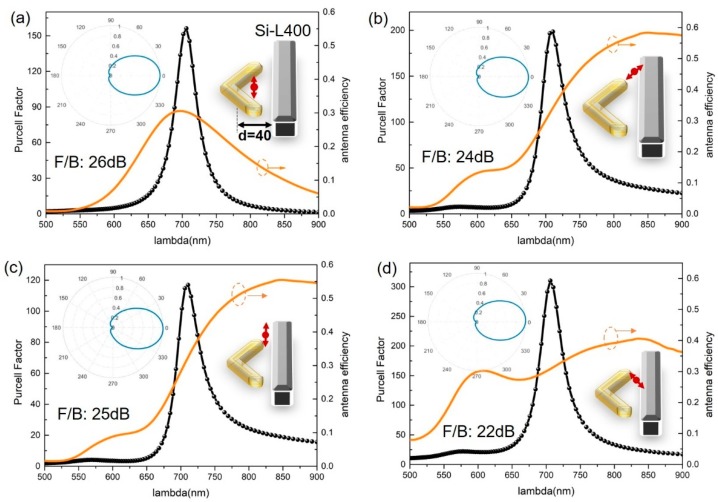
Performances of hybrid antenna designs for a single-element V-shape plasmonic dipole nanoantenna. Unidirectional emissions were all well-achieved for a V-shape nanoantenna (with an arm length of 60 nm and an open angle of 120°) in many emitter–antenna configurations. The strong coupling was evidenced by the large PF and high antenna efficiency (**a–d**). The black curves in all the figures are the PFs and the orange curves plot the antenna efficiency. The insets in each panel highlight the configurations of a localized emitter coupled with the hybrid antenna and the corresponding polar plot of the far-field radiation pattern. For all the calculations, the emitter was placed 10 nm away from the gold structure.

To this end, it is evident that hybrid antennas preserve high forward directivity with a broadband operating wavelength, which facilitates more versatile antenna designs. As such, a large library of plasmonic dipole antennas can be incorporated with their powerful strength in radiative decay enhancement. In the meantime, the strong redirection of emissions accomplished by the nearby silicon nanorod can severely modify the detection efficiency. The key design principle for the hybrid antenna is rather straightforward. Once we have established the relationship of the F/B ratio as a function of the wavelength for different emitter–silicon nanorod separations (like we have already done in Figure 2c–f), the remaining job is to devise the plasmonic dipole antennas with the resonant wavelength identical to the optimal wavelength of unidirectionality. Then, place the plasmonic dipole antenna at the corresponding position relative to the silicon nanorod. More sophisticated optical antenna configurations can be constructed and are demonstrated in Figure 5.

We designed the single-element V-shaped gold nanoantenna with an arm length of 60 nm to only support the dipolar mode excited by a local emitter. Such a V-shaped nanoantenna has previously been proposed to provide an unidirectional emission [9]. However, this was based on the multipolar interference resulting from the local excitation that requires a considerably large size for the V-antenna (i.e., an arm length of 240 nm). Moreover, a previous report has shown that high directivity only occurs for emitters at certain, well-defined positions relative to the antenna. In our designs, the arm length of the gold V-antenna was four times smaller than the plasmonic V-antenna and the unidirectional emission remained for all the strong coupling emitter-antenna configurations, as shown in Figure 5. The results further confirm that the individual silicon nanorod behaving as a unidirectional auxiliary element is generally applicable for various dipolar emission systems.

Furthermore, we replaced the single plasmonic dipole antenna with the dimer antenna, taking advantage of the *hotspot effect* within the gap to achieve a stronger modification of the spontaneous emission rate together with further avoidance of non-radiative loss. Asymmetric plasmonic gap antennas possessing multi-resonant modes have been proposed to enhance the fluorescence and the nonlinear optical process [36,43,44]. We showed in our study that the hybrid antennas consisting of such gap antennas and the silicon nanorod are outstanding optical routers which spectrally sort the emissions from the local point source into distinct far-field directions, as well as possess a large emission gain introduced by the nanogap.

**Figure 6:**
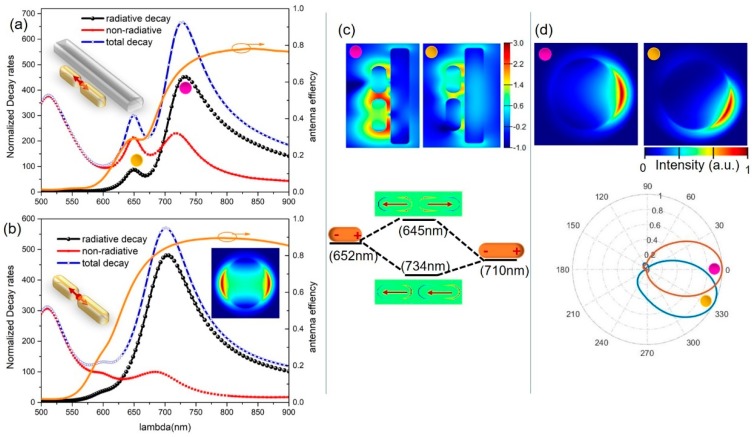
(**a**) Radiative, non-radiative, and total decay rates for hybrid antennas comprising a plasmonic gap antenna and a silicon nanorod (configuration shown in the inset), as well as the antenna efficiency (orange curve). Two distinct peaks corresponding to bonding and anti-bonding modes (indicated with the colored dots) are clearly observed in the decay rates curves. (**b**) Decay rates and antenna efficiency for the emitter in the middle of the gap antenna without a silicon nanorod. The BFP image in the inset clearly shows the omnidirectional dipolar feature. (**c**) Log-plot of the near-field distributions |E/E_0_|^2^ and the charge distributions under the excitation of the plane wave. These show the optical properties of bonding and antibonding modes caused by plasmon hybridizations. (**d**) Far-field radiation patterns for bonding and antibonding modes.

When the nanorod dimers (R40-L100 and R40-L120 with an end-to-end with gap of 20 nm) were excited with the nanoemitter located in the gap center, both the symmetric bonding and the asymmetric antibonding modes could be excited. It is shown in Figure 6a,b in the presence of the silicon nanorod. In addition to keeping the radiative decay rate (or PF) corresponding to the bonding mode nearly unaffected, the antibonding mode was apparently enhanced by the nearby silicon nanorod due to the increased effective refractive index. For the same reason, both the bonding and antibonding modes were spectrally red-shifted compared to those without silicon nanorods. Moreover, the nanocavity formed by the two gold nanorods further increased the antenna quantum efficiency compared to a single gold nanorod.

The dominant dipolar mode is the symmetric bonding mode at which the electric field in the gap region is remarkably enhanced, forming a so-called *hotspot*. Based on the near-field distribution shown in Figure 6c, it is evident that, when excited resonantly at the antibonding mode, the field enhancement appears less concentrated around the long rod and attains significant values near the short rod only. Notably, the silicon nanorod scatterer was able to redirect both resonant modes emission to the forward direction and spatially separate them very efficiently. The far-field BFP patterns for 734 nm (in the bonding mode) and 645 nm (in the antibonding mode) were calculated and are shown in Figure 6d. Interestingly, the sensitive angle resolving capability (also discussed in the context of Figure 3) of the beam redirection for the hybrid antenna system provides us with important information about the emission center at different resonant modes. In the bonding mode, the field enhancement lay in the vicinity of both rods and the emission center was located in the middle of the nanogap. Therefore, the emission direction was straight forward at a φ of 0°. In contrast, in the antibonding mode, the near-field was significantly distributed around the short rod, and the maximal φ of the angular pattern shifted to around 30°.

The observed optical routing property can contribute to a variety of applications. Plasmonic-enhanced fluorescence uses the concept of spectral features of multifrequency antennas covering both fluorophore absorption and emission to obtain maximal performance [45]. The plasmonic-enhanced nonlinear optical process uses the principle of multifrequency antennas with field enhancement at the fundamental frequency and Purcell factor enhancement at the conversion frequency [44]. Our designs pinpoint the hybrid nanoantennas ability to realize the spatially distinguishing up-conversion and down-conversion signals in energy exchange processes during light-matter interactions, which is beneficial in many practical applications.

## 4. Conclusions

In summary, a single dielectric silicon nanorod was used to efficiently rescatter the emission of a nearby electric dipole source. The interplay between the local excitation of the electric dipolar mode of the silicon nanorod and the dipole source was attributed to the high forward directivity, offering more parameters for optimizing the emission directivity than using classical metallic elements only. The unidirectional characteristics were also proven to be generally applicable for many typical plasmonic dipole antennas, such as nanorods, single element V-shape nanoantennas, and gap plasmonic nanoantennas. Without the use of bulky optical components, the hybrid antenna was ultracompact, with subwavelength dimensions of 0.08 λ^2^, and it operated in the visible spectral range. The antenna design combined advances in the fields of radiative decay engineering and signal routing to realize a new type of hybrid optical antennas. This will lead to more control of light in receiving and transmitting optical signals at the nanoscale.
